# Integration of Bulk and Single-Cell Transcriptomics Reveals Prognostic and Immunological Roles of MTHFD2 in Clear Cell Renal Cell Carcinoma

**DOI:** 10.3390/ijms27042021

**Published:** 2026-02-20

**Authors:** Yang Zhou, Xinmin Zheng, Penghui Ye, Hui Yang

**Affiliations:** 1 School of Life Science and Technology, Northwestern Polytechnical University, Xi’an 710072, China; drzhouyang@163.com (Y.Z.); zxm19891223@mail.nwpu.edu.cn (X.Z.); ye@mail.nwpu.edu.cn (P.Y.); 2 Engineering Research Center of the Chinese Ministry of Education for Biological Diagnosis, Treatment and Protection Technology and Equipment, Northwestern Polytechnical University, Xi’an 710072, China

**Keywords:** clear cell renal cell carcinoma, MTHFD2, single-cell transcriptomics, tumor-associated macrophages, prognostic biomarker, immune microenvironment, cell–cell communication, macrophage polarization

## Abstract

Tumor-associated macrophages (TAMs) are pivotal in the clear cell renal cell carcinoma (ccRCC) microenvironment. Methylenetetrahydrofolate dehydrogenase 2 (MTHFD2), a central enzyme in one-carbon metabolism, is increasingly recognized for its oncogenic roles in both cancer cells and immune compartments. We integrated bulk and single-cell transcriptomic datasets to interrogate the expression, prognostic impact, and immunomodulatory landscape of MTHFD2 in ccRCC. Robust differential expression, meta-analysis, Cox regression, and cell type deconvolution were performed. MTHFD2 expression and its association with prognosis were validated using tissue microarrays (TMAs), multiplex IHC, and in vitro macrophage polarization assays. MTHFD2 was upregulated in ccRCC tumors and associated with poor prognosis across multiple cohorts. High MTHFD2 expression remained an independent prognostic marker after adjustment for clinical stage. Single-cell analyses identified macrophages as the principal immune subpopulation expressing MTHFD2, with MTHFD2+ macrophages displaying a transcriptional signature of immunosuppression and metabolic adaptation. In vitro, MTHFD2-induced M2 macrophage polarization was reversed by DS18561882, promoting M1 polarization. MTHFD2 is a robust biomarker for poor prognosis in ccRCC, influencing tumor–immune interactions and macrophage polarization. Targeting MTHFD2 may represent a dual-action strategy to suppress tumor growth and reprogram the tumor immune microenvironment.

## 1. Introduction

Tumor-associated macrophages (TAMs) are a heterogeneous and plastic population deeply embedded in the tumor microenvironment of clear cell renal cell carcinoma (ccRCC), orchestrating processes such as angiogenesis, immune evasion, extracellular matrix remodeling, and metastasis [1,2,3]. TAMs are recruited both from circulating monocytes and tissue-resident sources and adapt their phenotype in response to microenvironmental cues such as hypoxia and cytokine signaling [4,5,6,7]. The classic M1/M2 dichotomy is oversimplified; in ccRCC, TAMs frequently exhibit an M2-like or hybrid state, marked by production of growth factors and immunoregulatory molecules that favor tumor progression [2,8].

Single-cell RNA-seq studies have resolved at least several functional macrophage subpopulations within the ccRCC microenvironment, each with unique transcriptional and functional properties, supporting angiogenesis, matrix remodeling, and active signaling with tumor cells and stromal elements [1,3,9,10,11]. Marker analysis has linked M2-associated markers (e.g., CD163, CD206) to poor clinical outcomes and immune suppression [2,3]. Beyond secretory and remodeling roles, TAMs directly interact with ccRCC cells via ligand–receptor pairs (such as EGF-CSF1 and OSM-OSMR), promoting epithelial-to-mesenchymal transition, invasion, and metastasis [8,12,13,14,15]. Notably, a recent single-cell study identified a TREM2+ macrophage subpopulation whose infiltration serves as a potential prognostic biomarker for post-surgical recurrence in ccRCC [16].

MTHFD2, a mitochondrial enzyme crucial for one-carbon (1C) metabolism, has recently emerged as a regulator not only of tumor cell proliferation and metabolic adaptation, but also as a functional and metabolic checkpoint in immune cells, including macrophages [17,18]. In tumors, MTHFD2 is overexpressed, supporting nucleotide biosynthesis, epigenetic regulation, and cell cycle progression [17,19,20].

In ccRCC tumor cells, MTHFD2 overexpression supports proliferation, migration, and adaptation to hypoxic stress—partially through the metabolic support of HIF-2α mRNA methylation and translation, activating glycolysis and remodeling cellular energetics [7,8]. High MTHFD2 levels associate with advanced grade, larger tumor size, and poorer survival in clinical studies [21].

Collectively, these findings implicate MTHFD2 as a central molecular node coupling tumor metabolism to immune microenvironment modulation. This study utilizes integrated bulk and single-cell transcriptomic analyses to comprehensively define the prognostic and immunological roles of MTHFD2 in ccRCC, with particular attention to the function of MTHFD2+ macrophages and their communication networks.

## 2. Results

### 2.1. MTHFD2 as a Robust Diagnostic and Prognostic Biomarker in ccRCC

**Figure 1:**
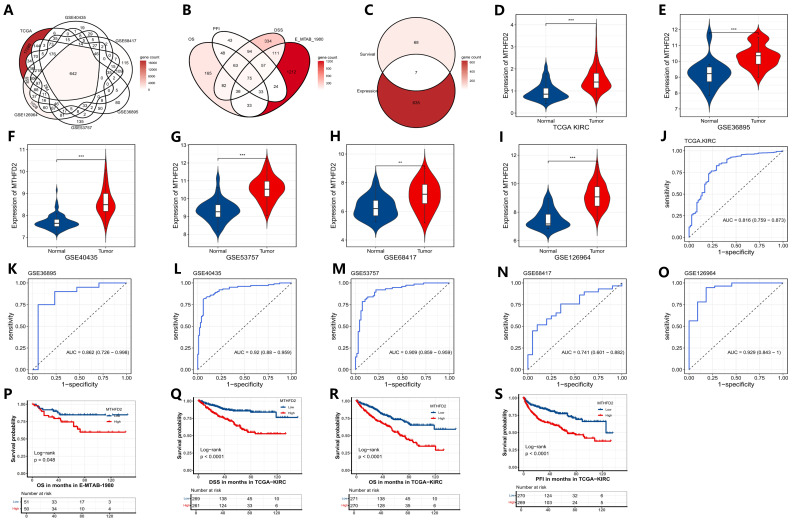
Identification and validation of MTHFD2 as a diagnostic and prognostic marker in ccRCC. (**A**) Overlap of upregulated DEGs across ccRCC datasets. (**B**) Venn diagram of prognosis-related genes across survival endpoints (OS, PFI, DSS). (**C**) Intersection identifying MTHFD2 as a consistently adverse marker. (**D**–**I**) Violin plots showing MTHFD2 expression in tumor versus normal tissues across datasets. (**J**–**O**) ROC curves assessing the diagnostic accuracy of MTHFD2 in distinguishing tumors from normal tissues. (**P**–**S**) Kaplan–Meier curves for OS, PFI, DSS in E-MTAB-1980 and TCGA, demonstrating poor survival with high MTHFD2 expression. Statistical markers: ** *p* < 0.01; *** *p* < 0.001.

Through integrative analysis of six bulk transcriptomic datasets, a set of robustly overlapping differentially expressed genes was identified in clear cell renal cell carcinoma (ccRCC), as illustrated in Figure 1A. By jointly evaluating associations with OS (overall survival), PFI (progression-free interval), and DSS (disease-specific survival) across cohorts, the selection of prognostically adverse genes was refined to maximize clinical relevance and robustness (Figure 1B). Within this intersection, MTHFD2 was distinguished as a gene that was not only consistently upregulated in ccRCC but also strongly correlated with unfavorable patient outcomes (Figure 1C), suggesting a pivotal role in disease progression.

Subsequent analysis revealed that MTHFD2 expression was significantly elevated in tumor tissues relative to adjacent normal tissues in every dataset examined, as demonstrated in Figure 1D–I. Receiver operating characteristic (ROC) curve analysis further confirmed the strong diagnostic potential of MTHFD2, with high area under the curve (AUC) values indicative of excellent discriminatory ability between tumor and normal samples (Figure 1J–O). Importantly, Kaplan–Meier survival analysis demonstrated that high levels of MTHFD2 expression were associated with shorter OS, PFI, and DSS in the TCGA cohort, as well as with poorer OS in the E-MTAB-1980 cohort (Figure 1P–S). Taken together, these results highlight MTHFD2 as a robust biomarker for both diagnosis and prognosis in ccRCC, providing a foundation for future studies exploring its value in clinical risk stratification and targeted therapies.

### 2.2. Independent Prognostic Value and Clinical Integration of MTHFD2 in ccRCC

**Figure 2:**
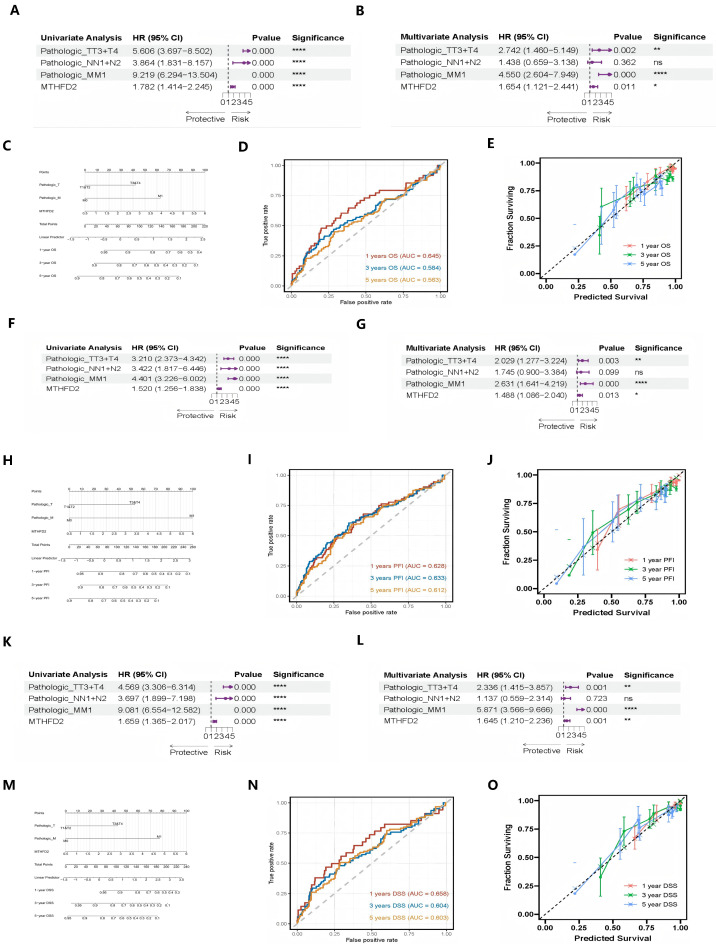
Independent prognostic value of MTHFD2 in ccRCC. (**A**) Univariable Cox forest plot showing associations of MTHFD2 and clinicopathological variables with OS. (The arrows (→) indicate that the upper bound of the 95% confidence interval (CI) extends beyond the displayed numerical range. This suggests that the true value could be higher than the maximum shown, reflecting substantial uncertainty or a wide confidence interval for these estimates.) (**B**) Multivariable Cox analysis confirming independent prognostic significance of MTHFD2. (**C**) Nomogram combining MTHFD2 expression and clinical parameters for survival prediction. (**D**) Time-dependent ROC curves for 1-, 3-, and 5-year OS prediction. (The black dashed line represents the reference line (AUC = 0.5), indicating no discrimination ability (equivalent to random chance). Curves above this line suggest better-than-random predictive ability.) (**E**) Calibration curves assessing nomogram accuracy on OS. (The black dashed line represents the ideal calibration line (perfect calibration), where predicted probabilities perfectly match observed outcomes. Perfect calibration would result in all points falling along this 45-degree diagonal line). (**F**–**J**) Similar analyses for PFI, confirming prognostic power. (**K**–**O**) Parallel analyses for DSS validating MTHFD2 as an independent adverse factor. Statistical markers: * *p* < 0.05; ** *p* < 0.01; **** *p* < 0.0001; ns = not significant.

Univariable Cox regression analysis demonstrated that MTHFD2 expression, along with traditional clinicopathological parameters including tumor stage (T), nodal status (N), and metastatic status (M), was significantly associated with overall survival (OS) in clear cell renal cell carcinoma (Figure 2A). To further delineate the independent prognostic utility of MTHFD2, multivariable Cox regression was performed, adjusting for T, N, and M classification. Notably, MTHFD2 remained a statistically significant adverse prognostic factor (hazard ratio > 1, *p* < 0.01) after controlling for these established variables (Figure 2B), demonstrating its robustness as an independent molecular predictor of outcome. These findings highlight the added value of integrating MTHFD2 expression into conventional clinical risk models, supporting its potential application in individualized prognostic assessment and precision oncology strategies for ccRCC.

Building upon this, nomograms integrating MTHFD2 expression with traditional clinicopathological variables were developed to provide individualized risk predictions (Figure 2C). Time-dependent receiver operating characteristic (time-ROC) analysis demonstrated that MTHFD2 expression provides robust prognostic discrimination for overall survival at 1-, 3-, and 5-year timepoints (Figure 2D). Furthermore, calibration analysis revealed strong agreement between nomogram-predicted and observed survival outcomes at all timepoints, indicating excellent model reliability (Figure 2E). Collectively, these results validate the potential of MTHFD2 expression as a clinically valuable parameter for risk stratification and survival prediction in patients with clear cell renal cell carcinoma.

Consistent with the OS findings, parallel analyses for PFI (Figure 2F–J) and DSS (Figure 2K–O) yielded concordant results. MTHFD2 expression retained its prognostic significance across all survival endpoints after multivariable adjustment, reinforcing its value as a robust and independent predictor of adverse clinical outcomes in clear cell renal cell carcinoma.

### 2.3. Functional Network and Biological Pathways Associated with MTHFD2 in ccRCC

**Figure 3:**
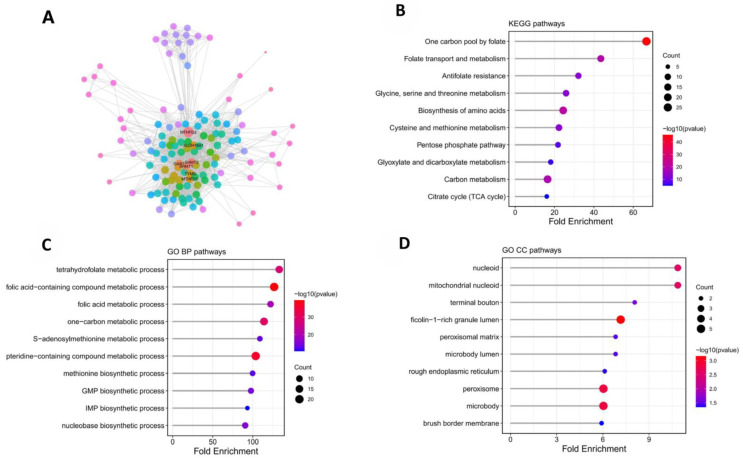
PPI network and enrichment analysis for MTHFD2-associated functional context. (**A**) STRING-based protein–protein interaction network of MTHFD2 and its interacting partners. (Node colors in the PPI network indicate the degree (number of direct interactions) of each protein. Nodes with higher degree values are highlighted in red, while nodes with lower degree values are shown in cooler colors.) (**B**) KEGG pathway enrichment identifying one-carbon metabolism, nucleotide synthesis, and energy pathways. (**C**) GO-BP enrichment of MTHFD2-related proteins, highlighting mitochondrial and biosynthetic processes. (**D**) GO-CC enrichment of MTHFD2-related proteins, highlighting mitochondrial nucleoid.

Protein–protein interaction (PPI) network analysis revealed that MTHFD2 is closely associated with a range of mitochondrial and biosynthetic protein partners, as illustrated in Figure 3A. These interacting proteins underscore the critical role of MTHFD2 within the mitochondrial matrix, where it serves as a central node linking various enzymatic systems involved in cellular metabolism and biosynthesis. To further elucidate the functional landscape of the MTHFD2 interactome, enrichment analysis using Gene Ontology (GO) and Kyoto Encyclopedia of Genes and Genomes (KEGG) databases was performed on the PPI network. The results demonstrated significant enrichment in biological processes and pathways related to one-carbon metabolism, nucleotide biosynthesis, and diverse aspects of mitochondrial function, including energy production and metabolic regulation (Figure 3B–D). Taken together, these findings comprehensively delineate the molecular context in which MTHFD2 operates, highlighting its integral involvement in fundamental metabolic pathways that support tumor growth and survival in ccRCC.

### 2.4. Transcriptomic Signature and Pathway Enrichment Associated with MTHFD2 Expression in ccRCC

**Figure 4:**
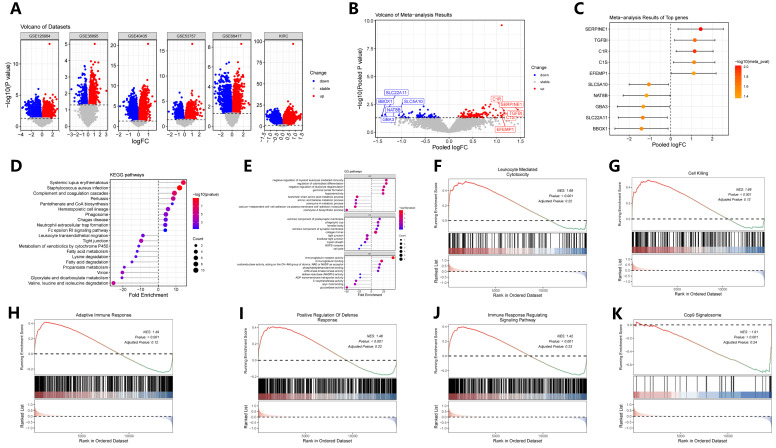
Meta-analysis of MTHFD2-associated transcriptomic signatures. (**A**) Volcano plots of DEGs between high- and low-MTHFD2 expression tumors across individual datasets. (**B**) Pooled meta-analysis volcano plot showing combined differential expression results. (**C**) Forest plot summarizing top meta-analytic DEGs with direction and effect size. (**D**) KEGG pathway enrichment of pooled DEGs. (**E**) GO biological process enrichment highlighting metabolic and immune pathways. (**F**–**K**) Representative GSEA plots for selected gene sets. The red-to-blue color bar represents the ranked gene list, where red indicates up-regulation and blue indicates down-regulation.

A meta-analysis of pooled differential expression comparing tumors with high versus low MTHFD2 expression uncovered a robust set of genes that were consistently upregulated or downregulated across multiple datasets (Figure 4A–C). This comprehensive differential gene signature provided a molecular landscape tightly linked to the stratification of MTHFD2 expression within ccRCC. Subsequent pathway enrichment analysis, utilizing GO, KEGG, and Gene Set Enrichment Analysis (GSEA), revealed that the MTHFD2-regulated gene signature was significantly enriched in biological pathways related to various metabolic processes and immune modulation (Figure 4D–K). Specifically, upregulated genes in the high MTHFD2 group were involved in immune cell-related pathways, while downregulated genes included elements associated with fatty acid and amino acid metabolism functions. Collectively, these findings underscore the central role of MTHFD2 in driving a transcriptomic program that promotes metabolic and immunomodulatory adaptations conducive to tumor aggressiveness in ccRCC.

### 2.5. Immunological Landscape and Functional Heterogeneity of Macrophages Associated with MTHFD2 in ccRCC

**Figure 5:**
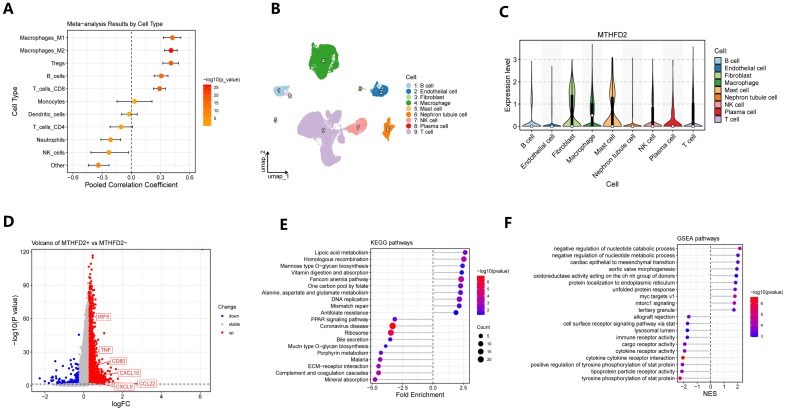
Association of MTHFD2 with immune infiltration and macrophage heterogeneity. (**A**) Meta-analysis forest plot showing the pooled correlations between MTHFD2 expression and the infiltration of various immune cell types across cohorts. (**B**) UMAP visualization of single-cell transcriptomes displaying the major cell types in ccRCC. (**C**) Violin plots showing MTHFD2 expression across distinct cellular populations. (**D**) Volcano plot of differentially expressed genes between MTHFD2+ and MTHFD2- macrophages. (**E**) KEGG pathway enrichment analysis of upregulated genes in MTHFD2+ macrophages, revealing enrichment of immune regulatory and metabolic pathways. (**F**) GSEA enrichment confirming enhanced metabolic and immunomodulatory functions in MTHFD2+ macrophages.

Comprehensive analysis of the immunological infiltration in ccRCC revealed a strong positive correlation between MTHFD2 expression and the degree of M1 macrophage infiltration, as determined by meta-analytical integration across multiple datasets (Figure 5A). This relationship suggests that tumors with elevated MTHFD2 expression are characterized by a higher abundance of tumor-associated macrophages (TAMs), hinting at a potential interplay between metabolic reprogramming and immune cell recruitment. Single-cell RNA sequencing data, visualized using UMAP projections and violin plots, further demonstrated that MTHFD2 expression was predominantly elevated not only in macrophages but also in fibroblasts and in mast cells within the tumor microenvironment (Figure 5B,C), reflecting its selective upregulation among key stromal and immune cell compartments. Within the macrophage compartment, stratification of TAMs based on MTHFD2 expression status revealed that MTHFD2-positive macrophages exhibited a distinct gene expression signature enriched for pathways involved in immune regulation, inflammatory responses, and altered metabolic processes (Figure 5D–F). This subset of macrophages displayed an upregulation of genes associated with immunosuppression, metabolic adaptation, and pro-tumorigenic functions, suggesting that MTHFD2 may mark a specialized macrophage population that actively contributes to shaping the tumor’s immune microenvironment and promoting tumor progression. Collectively, these findings emphasize the integral role of MTHFD2 in orchestrating macrophage heterogeneity and immune contexture in ccRCC, revealing novel insights into its potential as a target for therapeutic intervention aimed at modulating both metabolic and immune pathways.

### 2.6. Single-Cell Dissection of Cell–Cell Communication Reveals MTHFD2+ Macrophages as Central Signal Integrators in ccRCC

**Figure 6:**
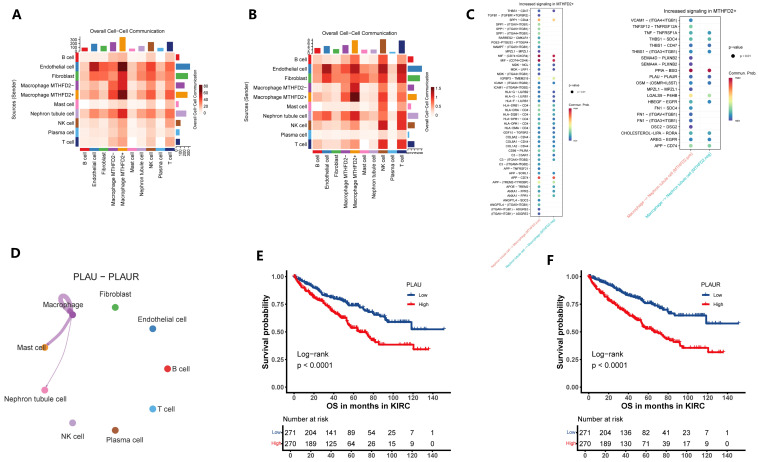
MTHFD2+ macrophages serve as key communication hubs within the ccRCC tumor microenvironment. (**A**,**B**) Heatmap summarizing ligand–receptor interaction numbers among major cell types. (**C**) Bubble plot of major signaling pathways from epithelial cells to macrophages (Epi → Macro) and that from macrophages to epithelial cells (Macro → Epi). (**D**) Circle network diagram illustrating the overall increase in PLAU-PLAUR communication involving MTHFD2+ macrophages. (**E**,**F**) Kaplan–Meier curves showing that elevated expression of the ligand–receptor pair PLAU and PLAUR predicts poorer overall survival in ccRCC patients.

Single-cell RNA sequencing analysis enabled the precise identification of MTHFD2-positive macrophages as a distinct subpopulation within the tumor immune microenvironment of ccRCC. These MTHFD2+ TAMs were subjected to further investigation through CellChat network analysis, which revealed their role as prominent nodal cells in the landscape of intercellular communication, especially through paracrine interactions with nephron epithelial tumor cells (Figure 6A,B). Detailed examination of the reconstructed signaling networks indicated that MTHFD2+ TAMs engage in significantly enhanced ligand–receptor-mediated signal exchange compared to their MTHFD2-negative counterparts, with increased communication observed in both directions—that is, from epithelial cells to macrophages (Epi → Macro) as well as from macrophages to epithelial cells (Macro → Epi). These alterations in cellular crosstalk were characterized by the upregulation of key ligand and receptor genes that participate in pathways associated with immune modulation, inflammation, and tumor progression (Figure 6C).

Focusing on specific signaling events, CellChat-based ligand–receptor analysis identified the PLAU-PLAUR pair as markedly upregulated within MTHFD2+ TAMs and predominantly engaged in communication with epithelial tumor cells (Figure 6D). Survival analyses demonstrated that elevated expression of both PLAU and PLAUR was significantly correlated with poor patient outcomes, highlighting their potential role in mediating malignant progression (Figure 6E,F). Collectively, these findings suggest that MTHFD2+ TAMs may promote tumor aggressiveness in ccRCC through activation of suggest a potential mechanism whereby MTHFD2+ TAMs could contribute to tumor attressiveness in ccRCC by engaging the PLAU-PLAUR signaling axis within the tumor microenvironment. Overall, single-cell mapping of these cellular communication networks underscores the influential role of MTHFD2+ macrophages as central orchestrators of the paracrine signaling landscape in ccRCC, offering novel avenues for therapeutic targeting of the macrophage–tumor cell axis.

### 2.7. Validation of MTHFD2 Overexpression and Clinical Prognostic Value in ccRCC

**Figure 7:**
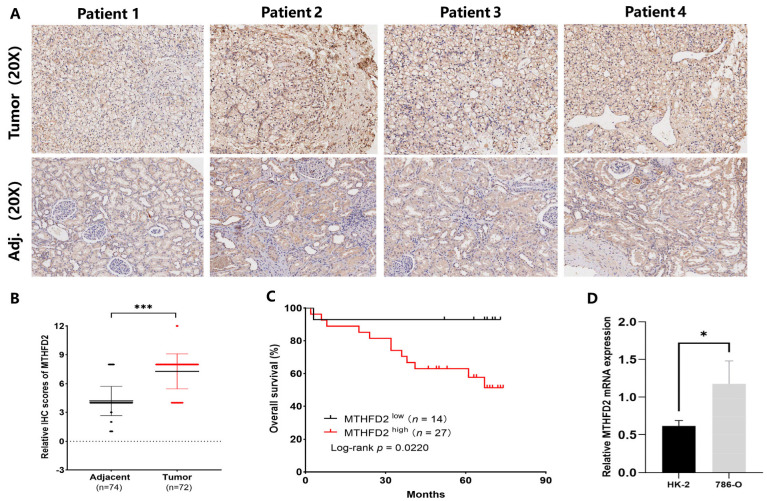
Validation of MTHFD2 overexpression and its prognostic relevance in ccRCC. (**A**) Representative IHC images showing MTHFD2 protein expression in tumor and adjacent normal tissues. (**B**) Quantitative comparison of IHC staining scores demonstrating MTHFD2 upregulation in tumors. (**C**) Kaplan–Meier survival analysis showing reduced overall survival in the high-MTHFD2 group (H-score ≥ 8). (**D**) RT-qPCR confirming higher MTHFD2 mRNA levels in 786-O renal carcinoma cells than in normal HK-2 epithelial cells. Statistical markers: * *p* < 0.05; *** *p* < 0.001.

IHC analysis of the patient TMA from cohort 1 confirmed that MTHFD2 was indeed highly expressed in ccRCC tissues, with quantitative evaluation further validating this elevation compared with adjacent normal samples (Figure 7A,B). Kaplan–Meier analysis showed that high MTHFD2 expression (H-scores ≥ 8) was significantly associated with reduced overall survival (*p* = 0.022) (Figure 7C). Additionally, MTHFD2 mRNA levels were significantly higher in 786-O RCC cells than in HK-2 cells (*p* = 0.034) (Figure 7D). These findings indicate that MTHFD2 plays a role in ccRCC and that elevated MTHFD2 levels are linked to an unfavorable prognosis in this malignancy.

### 2.8. MTHFD2 Is Positively Correlated with CD68 and CD163 Expression and Induces Macrophage Polarization in ccRCC

**Figure 8:**
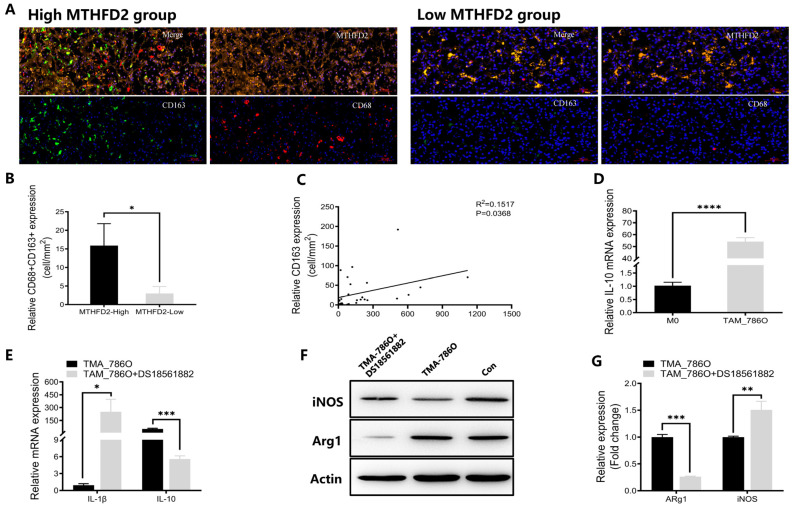
MTHFD2 promotes macrophage infiltration and M2 polarization in ccRCC. (**A**) Multiplex IHC images showing co-staining of MTHFD2, CD68, and CD163 in ccRCC tissues. (**B**) Quantitative analysis of CD68 + CD163+ macrophage infiltration between MTHFD2-high and MTHFD2-low groups. (**C**) Correlation analysis between MTHFD2 and CD163 expression across the ccRCC cohort. (**D**) RT-qPCR analysis of IL-10 expression in macrophages co-cultured with 786-O cells, confirming M2-type polarization. (**E**) RT-qPCR analysis of IL-1β and IL-10 mRNA expression in THP-1-derived macrophages after treatment with the MTHFD2 inhibitor DS18561882 (150 nM). (**F**,**G**) Western blot analysis of iNOS and Arg1 protein expression in THP-1-derived macrophages with and without DS18561882 treatment (150 nM). Statistical markers: * *p* < 0.05; ** *p* < 0.01; *** *p* < 0.001; **** *p* < 0.0001.

To validate the relationship between MTHFD2 expression and macrophage infiltration, we conducted multiplex IHC staining of MTHFD2, CD68, and CD163 in ccRCC tissues in the TMA of cohort 2. CD68 and CD163 were more co-expressed in tumor tissues with high MTHFD2 expression than in tumor tissues with low MTHFD2 expression (*p* = 0.0412) (Figure 8A,B). Additionally, there was a significant association between MTHFD2 expression and CD163 expression (R^2^ = 0.1517, *p* = 0.0368), and the latter represents the degree of M2-polarized macrophage infiltration (Figure 8C).

The regulatory role of MTHFD2 in TAMs was evaluated in vitro. Human THP-1 monocytes were treated with PMA for 24 h to induce differentiation into M0 macrophages. Then, M0 macrophages were co-cultured with 786-O RCC cells for 48 h to generate TAMs. The RT-qPCR results demonstrated that the levels of IL10, an M2 marker, increased in TAMs (Figure 8D), indicating the generation of M2-polarized macrophages in vitro. Additionally, RT-qPCR and Western blot analyses revealed that a dose (150 nM) of DS18561882 reduced the expression of M2 markers (IL-10 and Arg1) and increased the expression of M1 markers (IL-1β and iNOS) in TAMs co-cultured with 786-O cells (Figure 8E–G). These findings suggest that MTHFD2 induces M2 polarization of TAMs in ccRCC.

## 3. Discussion

The overexpression of MTHFD2, a key enzyme in folate metabolism, has been implicated in various malignant features and is associated with adverse prognosis in multiple cancers [22]. In our study, our integrative transcriptomic analysis revealed that MTHFD2 is consistently overexpressed in ccRCC tumors and correlates strongly with poor patient outcomes, even after adjusting for established clinicopathological parameters. IHC and RT-qPCR analyses confirmed the overexpression of MTHFD2 in ccRCC tissue microarrays (TMAs) and 786-O RCC cells. Moreover, IHC scores were positively correlated with poorer prognosis in ccRCC. These findings are consistent with previous studies indicating that elevated MTHFD2 expression predicts adverse outcomes in ccRCC [19,20] further validating the potential of MTHFD2 as a reliable clinical marker.

Additionally, our findings extend the current understanding by linking MTHFD2 expression not only to tumor cells but also to the immune microenvironment, particularly macrophages. Single-cell RNA sequencing and protein–protein interaction analyses revealed that MTHFD2 expression is prominently upregulated in tumor-associated macrophages (TAMs), which are known to contribute to immunosuppression and tissue remodeling within the tumor microenvironment [6,23]. Our data reveal that macrophages represent a principal non-neoplastic population with high MTHFD2 expression, and within this compartment, MTHFD2 stratifies immune-regulatory state, reflecting a shift toward immunosuppressive and metabolically adaptive phenotypes. These findings underscore the dual role of MTHFD2 in both tumor cells and immune cells, positioning it as a central node in the tumor microenvironment. Specifically, MTHFD2+ macrophages are not only enriched in adverse prognostic environments but also actively participate in tailored cell–cell communication programs directing pro-tumoral signaling circuits with epithelial cells. Ligand–receptor analyses reinforce a model in which metabolic reprogramming of macrophages—driven by MTHFD2—remodels both immune resistance and tumor-microenvironmental cross-talk. Recent reports have indicated that MTHFD2 supports the metabolic shifts required for macrophage differentiation into immunosuppressive M2-like phenotypes, further enhancing their pro-tumoral activities [22,24].

Furthermore, multiplex IHC staining of ccRCC TMA revealed that CD68 and CD163, markers of macrophage polarization, were co-expressed in tumor tissues with high MTHFD2 expression. A strong association was observed between MTHFD2 and CD163 levels, implicating MTHFD2 in macrophage polarization. Moreover, the MTHFD2 inhibitor DS18561882 reversed the polarization of TAMs co-cultured with 786-O RCC cells, suggesting that MTHFD2-driven macrophage polarization may contribute to tumorigenesis or aggressiveness. This study is the first to demonstrate the role of MTHFD2 in ccRCC progression through macrophage polarization. However, further research is needed to clarify the molecular pathways underlying the effect of MTHFD2 on macrophages and tumor progression. Notably, our in vitro co-culture experiments utilized only a single RCC cell line (786-O); incorporating additional cell lines in future studies would help to validate and generalize these findings.

In conclusion, our study underscores the multifaceted role of MTHFD2 in ccRCC, where it functions as a key mediator of tumor metabolism and immune modulation. By driving metabolic reprogramming in both tumor cells and macrophages, MTHFD2 contributes to the aggressive nature of ccRCC and promotes a tumor-supportive immune environment. These findings support the potential of MTHFD2 as both a biomarker for poor prognosis and a therapeutic target in ccRCC. Further studies are warranted to explore the feasibility of MTHFD2 inhibition as a dual-action strategy to target tumor growth and reprogram the immune landscape in ccRCC.

## 4. Materials and Methods

### 4.1. Data Collection and Processing Bulk Transcriptomics

Bulk transcriptomic data were collected from TCGA-KIRC and multiple GEO datasets (GSE40435, GSE68417, GSE36895, GSE53757, GSE126964). For microarray data, probe-to-gene mapping was performed using annotation files, and gene expression values were normalized within platforms. Differential expression analysis was carried out using DESeq2 for RNA-seq data to compare tumor and normal samples [25]. For microarray data, the limma package (v3.66.0) with empirical Bayes moderation was used for the tumor versus normal contrast, and genes meeting the criteria of *p* < 0.05 and log2 fold-change larger than 1.5 were retained for further analysis [26]. Finally, gene SYMBOLs were mapped to ENTREZ IDs using the org.Hs.eg.db (v3.14.0) and clusterProfiler packages (v4.18.2) for downstream analyses [27].

### 4.2. Survival Analysis

Survival analysis was performed using the TCGA-KIRC and E-MTAB-1980 cohorts, applying univariable Cox regression for overall survival (OS), progression-free interval (PFI), and disease-specific survival (DSS), with gene expression dichotomized at the median; genes were considered significant if they had a *p* value less than 0.05 and a hazard ratio greater than 2.

### 4.3. Single-Cell Transcriptomic Analysis

Single-cell transcriptomic datasets GSE111360 and GSE159115 were processed by first importing the 10x matrices into Seurat (v4.3.0) and removing non-ccRCC samples. Quality control criteria included filtering cells with nFeature_RNA between 200 and 5000, mitochondrial gene percentage below 15%, ribosomal gene percentage below 40%, and nCount_RNA between 500 and 20,000. Decontamination was performed using decontX, and doublets were excluded with scDblFinder [28,29]. Subsequent steps involved batch integration using FastMNN, followed by dimensionality reduction through UMAP or tSNE, and final cell-type classification was conducted using scGate with the HiTME model.

### 4.4. Meta-Analysis

For the meta-analysis of gene differential expression across multiple datasets, we first identified genes that were commonly detected in all datasets through intersecting gene symbol lists. For each such gene, results from individual studies were compiled into a unified table containing gene symbols, log fold changes, standard errors (calculated as the square root of the posterior variance from the limma output), and *p*-values. Meta-analysis of differential expression was conducted for each gene with data available from at least two studies, using a random-effects model (REML estimation). For each gene, the model yielded pooled log fold changes, standard errors, *p*-values, study counts, and measures of heterogeneity. The combined meta-analytic results were then merged, and significance was assigned according to an FDR threshold adjusted by the Benjamini–Hochberg method. Genes were further categorized as upregulated, downregulated, or stable based on the direction and significance of the pooled effect. Confidence intervals were calculated as logFC ± 1.96 × SE.

To perform a meta-analysis of correlation coefficients between gene expression and immune cell infiltration across different studies, we first collected output lists containing correlation coefficients and *p*-values from quanTIseq analysis for each dataset [28]. For each cell type, the results from all studies were merged into a unified data structure, annotating the study source and categorizing statistical significance based on a *p*-value threshold of 0.05. To enable meta-analytic pooling for each immune cell type, Spearman correlation coefficients were approximately converted to Pearson correlation coefficients and then transformed using Fisher’s z-transformation, with standard errors calculated as the reciprocal of the square root of sample size minus three. Random-effects models were fitted using the REML method (rma function in the metafor package) (v4.8.0), providing pooled effect size estimates, confidence intervals, heterogeneity statistics (I^2^ and τ^2^), and *p*-values. The pooled summary measures and their confidence intervals were then converted back to the correlation scale. This meta-analysis was performed independently for each immune cell type by grouping the data accordingly and extracting relevant summary statistics, which were then ranked by the absolute value of the pooled correlation coefficient.

### 4.5. Protein–Protein Interaction Analysis

The direct protein–protein interaction (PPI) partners of MTHFD2 were identified using the STRING database, ensuring a comprehensive assessment of experimentally validated and predicted molecular associations [30]. The resulting interaction network was subsequently visualized and analyzed using the igraph (v1.5.1) and ggraph packages (v2.2.2), which enabled detailed exploration of the topological structure and relationship patterns among interacting proteins. To gain insights into the functional roles and biological pathways related to the MTHFD2 PPI subnetwork, Gene Ontology (GO) and Kyoto Encyclopedia of Genes and Genomes (KEGG) pathway enrichment analyses were performed.

### 4.6. Immune Cell Correlation

The abundance of various immune cell types within bulk transcriptomic datasets was estimated using quanTIseq deconvolution as implemented in the IOBR package (v0.99.8). For each dataset, a Spearman correlation analysis was performed to assess the association between MTHFD2 expression levels and the estimated abundance of individual immune cell populations. To ensure the robustness and generalizability of these associations across different platforms and cohorts, the resulting correlation coefficients from each dataset were subjected to meta-analysis, enabling the integration of results and the identification of consistent patterns of immune infiltration associated with MTHFD2 expression in ccRCC.

### 4.7. Macrophage Subtype and Functional Profiling (scRNA-Seq)

To further dissect the functional heterogeneity of tumor-associated macrophages, macrophage populations were extracted from the single-cell RNA-seq data and then stratified into MTHFD2-positive (MTHFD2+) and MTHFD2-negative (MTHFD2-) groups based on their MTHFD2 expression levels. Differential gene expression analysis between the MTHFD2+ and MTHFD2- macrophage subsets was performed using the FindMarkers function, enabling the identification of genes specifically upregulated or downregulated in association with MTHFD2 expression. The resulting marker genes from this comparison were subsequently subjected to Gene Ontology (GO), Kyoto Encyclopedia of Genes and Genomes (KEGG), and Gene Set Enrichment Analysis (GSEA) to elucidate the biological processes, pathways, and functional programs that distinguish MTHFD2+ macrophages from their MTHFD2- counterparts.

### 4.8. Cell–Cell Communication

Cell–cell communication analysis was conducted using the CellChat framework [31]. For this analysis, cells were categorized into three groups according to their identity: MTHFD2-positive macrophages, MTHFD2-negative macrophages, and non-macrophage populations. Communication networks were reconstructed separately for both the MTHFD2+ and MTHFD2- macrophage contexts to compare the differences in signaling patterns between these distinct subsets. Comprehensive visualizations—including heatmaps displaying the number and overall strength of ligand–receptor interactions, along with bubble plots and chord diagrams highlighting selected signaling axes—were generated to illustrate network complexity and identify key paracrine signaling routes. In order to determine the clinical relevance of the identified communication pathways, genes encoding differentially active ligands and receptors were cross-referenced with the TCGA-KIRC cohort, and survival analyses such as Kaplan–Meier plots and log-rank tests were performed.

### 4.9. Tissue Microarrays (TMAs) and Patient Cohorts

Two KIRC TMAs were purchased from Shanghai Outdo Biotech Company (Shanghai, China). Cohort 1 (HKid-CRCC150 CS-01) contained 75 cases of cancerous tissues and 75 cases of paracancerous tissues with OS information, and cohort 2 (HKidE030PG02-M-187) contained 30 cases of cancerous tissues without OS information. The study protocol was approved by the Shanghai Qutdo Biotech Co., Ltd. (Shanghai, China). Ethics Committee (HKid-CRCC 1SOCS-01, 1 July 2014, HKidE030PG02, 2 January 2015).

### 4.10. Immunohistochemistry (IHC)

The array underwent overnight incubation at 4 °C with rabbit polyclonal anti-MTHFD2 antibody (1:200, Proteintech, Wuhan, China), followed by washing with phosphate-buffered saline (PBS) and treatment with a biotin-conjugated secondary antibody. The TMA from cohort 1 was analyzed by IHC. The TMA was deparaffinized and processed for antigen retrieval. Next, the array was incubated overnight at 4 °C with rabbit polyclonal anti-MTHFD2 antibody (1:200, Proteintech, Wuhan, China), followed by washing with PBS, and treatment with a biotin-conjugated secondary antibody. After washing in PBS, the TMA was incubated with horseradish peroxidase (HRP) and visualized using diaminobenzidine (DAB) (Maixin Biotech, Fuzhou, China). Scores for immunohistochemical staining were calculated by combining the proportion of cells with positive staining as 0 (<10%), 1 (10–25%), 2 (25–50%), 3 (50–75%), and 4 (>75%) and the intensity of staining as 0 (none), 1 (weak), 2 (moderate), and 3 (strong). The overall scores varied between 0 and 12, with categories of low (<8) and high (≥8) being assigned.

### 4.11. Multiplex IHC Staining and Image Analysis

TMA 2 was analyzed by multiplex IHC. The AlphaTSA Multiplex IHC Kit (AXT37100031, Alpha X Biotech Co., Ltd., Beijing, China) was utilized for multicolor staining according to the manufacturer’s guidelines. The TMA was exposed to rabbit anti-MTHFD2 (1:800, Proteintech, China), mouse anti-CD68 (1:500, ZM0060, ZSGB-BIO, Beijing, China), and mouse anti-CD163 (1:200, ZM0428, ZSGB-BIO, Beijing, China). The slides were imaged on an Axioscan7 system (Carl Zeiss AG, Jena, Germany) running on ZEN software (v3.3). The number of cells positive for MTHFD2, CD68, and CD163 was calculated using AlphaPainter X30 (Alpha X Biotech Co., Ltd., Beijing, China) and was expressed as cells/mm [2,32].

### 4.12. Cell Lines and Culture

Human clear cell renal cell cancer 786-O cells, renal tubular epithelial HK-2 cells and THP-1 cells were purchased from cell Bank of the Chinese Academy of Sciences, China. Next, 786-O cells were grown in DMEM with 10% fetal bovine serum (FBS). THP-1 cells and HK-2 cells were grown in RPMI 1640 medium supplemented with 10% FBS at 37 °C in a 5% CO_2_ atmosphere.

### 4.13. Macrophage Generation and Differentiation

Prior to generating M0 macrophages, THP-1 cells were pre-incubated with 100 ng/mL of phorbol myristate acetate (PMA) (Med-ChemExpress, Monmouth Junction, NJ, USA) for 24 h. The M0 macrophages were then combined with 786-O cells in a 6-well transwell co-culture system (Corning Inc., Corning, NY, USA) with a pore size of 0.4 µm for a period of 48 h. Then, the co-cultured macrophages were collected to obtain tumor-associated macrophages (TAMs).

### 4.14. Drug Treatment

The MTHFD2 inhibitor DS18561882 was purchased from MedChemExpress (Monmouth Junction, NJ, USA). DS18561882 was dissolved in dimethyl sulfoxide (DMSO) to prepare a stock solution and further diluted in culture medium to the indicated working concentration. For in vitro experiments, tumor-associated macrophages (TAMs) generated by co-culture with 786-O cells were treated with DS18561882 at a final concentration of 150 nM, which was selected based on the product information and previously published in vitro studies demonstrating effective cellular inhibition of MTHFD2 [33]. An equal volume of DMSO was used as the vehicle control. After treatment, TAMs were collected for subsequent RT-qPCR and Western blot analyses to evaluate the expression of M1 (IL-1β and iNOS) and M2 (IL-10 and Arg1) macrophage polarization markers.

### 4.15. Western Blotting

After being washed twice with ice-cold PBS, the cells were then treated with RIPA reagent to prepare protein extracts. The levels of protein were measured with the Bradford method. 30 µg of proteins were separated using SDS-PAGE and then transferred to PVDF membranes. The membranes were probed with antibodies against MTHFD2 (rabbit, 1:1000′ Proteintech, China), ARg1, iNOS, and β-actin (1:5000, Proteintech, China). Immunoreactive bands were identified by enhanced chemiluminescence.

### 4.16. RNA Extraction and Real-Time Quantitative Polymerase Chain Reaction (RT-qPCR)

RNA was isolated with the RNeasy Mini Kit (Qiagen, Hilden, Germany) in accordance with the provided guidelines. RNA was converted into cDNA through reverse transcription with a high-capacity cDNA synthesis kit (Takara Bio Inc., Dalian, China). During the amplification process, a heating cycle was conducted at 95 °C for 3 min, then followed by 40 cycles at 95 °C for 6 s and 60 °C for 35 s. The expression levels of the specific genes were standardized based on β-actin expression using the 2ΔΔCt method. The primers for β-actin (forward, 5′-ACCCTGAAGTACCCCATCGAG-3′; reverse, 5′-AGCACAGCCTGGATAGCAAC-3′) and MTHFD2 (forward, 5′-GTGGATTTTGAAGGAGTCAG-3′; reverse, 5′-CTTTAGACTTCAGCACTTCTC-3′) were designed using Primer 10.0. The assays were performed in triplicate.

### 4.17. Statistical Analysis

Statistical analysis was conducted with GraphPad Prism 10.0 (GraphPad Software, San Diego, CA, USA) and R software version 4.1.2. Comparison between two groups was assessed with the Wilcoxon test, while distinctions among three or more groups were evaluated with the Kruskal–Wallis test. The relationships between genes and levels of immune cell infiltration were calculated using Pearson correlation analysis. All experiments conducted in vitro were replicated three times and performed in triplicate. Data are presented as mean values with standard errors. Statistical significance was defined as a *p*-value less than 0.05.

## 5. Conclusions

MTHFD2 serves as a diagnostic and prognostic marker in ccRCC, linking cellular metabolism to immune microenvironment modulation. As a regulated node in both tumor and macrophage compartments, MTHFD2 orchestrates transcriptional, metabolic, and paracrine programs underpinning tumor progression. Dual targeting of MTHFD2 within both lineages offers a compelling therapeutic approach to disrupt tumor growth and reinvigorate anti-tumor immunity in ccRCC.

## Data Availability

The original contributions presented in this study are included in the article. Further inquiries can be directed to the corresponding author.
